# Static scheduling method for aircraft flat-tail assembly production based on improved bi-level genetic algorithm

**DOI:** 10.1038/s41598-025-94027-9

**Published:** 2025-03-20

**Authors:** Tengda Li, Min Hua, Junliang Wang, Wei Qin

**Affiliations:** 1 Department of Industrial Energy Products, China Mobile Shanghai Industry Research Institute, Shanghai, 201206 China; 2 Shanghai Jiao Tong University, USC-SJTU Institute of Cultural and Creative Industry, Shanghai, 200240 China; 3 Institute of Artificial Intelligence, Donghua University, Shanghai, 201620 China; 4 Department of Industrial Engineering and Management, Shanghai Jiao Tong University, Shanghai, 200240 China

**Keywords:** Flat-tail assembly production, Flexible flow-shop scheduling problem, Improved bi-level genetic algorithm, Variable neighborhood search, Mechanical engineering, Computational science

## Abstract

Aircraft flat-tail assembly is a complex process that involves multiple assembly processes, multiple parallel frames, and multi-configuration mixed flow assembly, and its assembly processes exhibit extended processing times (typically measured in days) combined with temporal fluctuations arising from human factors, leading to a certain degree of uncertainty in single-process durations, thereby presenting a complicated flexible flow-shop scheduling problem (FFSP), which is a typical NP-hard problem. Despite its significance, the research on FFSP in aircraft flat-tail assembly production scheduling is limited. This study proposes an improved bi-level genetic algorithm to address the two sub-problems of flat-tail assembly production scheduling: frame assignment and assembly task sequencing. The objective is to minimize the maximum delay penalty cost. A two-stage coding scheme is introduced for frame assignment and task sequencing, respectively. To mitigate genetic algorithms’ convergence to local optima and enhance positive feedback, we implement a variable neighborhood search mechanism combined with elite retention. The efficacy of the improved bi-level genetic algorithm is evaluated through experiments and case studies in enterprises, indicating a significant impact on the assembly production scheduling of flat-tail, with potential applications to similar large and complex equipment. Overall, this study contributes to FFSP research in aircraft flat-tail assembly production scheduling by offering a novel solution approach to effectively address the sub-problems of frame assignment and assembly task sequencing.

## Introduction

Aircraft flat-tail assembly is a typical discrete assembly, and its assembly process has the following characteristics: (1) multiple assembly processes, the aircraft flat-tail passes through each assembly process in turn, which is a flow assembly operation; (2) multiple parallel assembly frames, each assembly process corresponds to an assembly station, and each assembly station has at least one assembly frame; (3) multi-configuration flat-tail mixed flow assembly, the same type of aircraft flat-tail has multiple configurations, and the assembly process of each configuration is the same, but there are differences in the assembly cycle time. Therefore, aircraft flat-tail assembly production scheduling is a typical Flexible Flow-shop Scheduling Problem (FFSP). However, compared to traditional FFSP problems, aircraft flat-tail assembly exhibits the following distinctive characteristics, including: (1) the assembly process involves simultaneous production of multiple configurations and orders, where different aircraft flat-tail configurations compete for shared resources including human resources, assembly frames, and auxiliary tooling; (2) the assembly frames for aircraft flat-tails represent substantial capital investments with limited availability, necessitating optimal scheduling of their utilization time and sequence; (3) the manual operation process, where workers collaborate with assembly equipment, introduces significant human-induced variability due to fluctuations in worker efficiency and unexpected interruptions, resulting in a certain uncertainty in task durations; (4) individual assembly operations exhibit extended processing times (typically measured in days) combined with temporal fluctuations arising from human factors, leading to a certain degree of uncertainty in single-process durations. Considering these unique manufacturing characteristics of aircraft flat-tail assembly, developing rational and effective scheduling strategies becomes crucial for: reducing assembly cycle time, improving on-time delivery capability for orders, enhancing customer satisfaction, and optimizing resource allocation in horizontal tail production systems. This research holds significant practical implications for advancing aircraft assembly efficiency and operational excellence.

Scholars in relevant fields have done extensive work on flexible flow-shop scheduling problems at present. Rakrouki et al.^1^ considered a problem of minimizing the total tardiness in a deterministic two-machine permutation flow-shop scheduling problem subject to release dates of jobs and known unavailability periods of machines. Devi et al.^2^ proposed a hybrid adaptive firefly algorithm to solve the flexible job shop scheduling (FJSP) problem. Adaptive parameters were embedded with the classic firefly algorithm to optimize the multi-objectives concurrently. Mousavi et al.^3^ investigated the problem of scheduling independent jobs in a g-stage hybrid flow shop environment with two additional traits. Mahdi and Lotfi^4^ investigated a two-stage hybrid flow-shop problem under setup times and proposed a metaheuristic using the genetic algorithm and three heuristics to solve it. Wang^5^ studied the problem of optimal scheduling of the flow shop, the machining sequence and maximum completion time were taken as the key points, a method of optimal scheduling by genetic algorithm (GA) was proposed, and a mathematical model for optimal scheduling of the flow shop based on GA was established. Isler et al.^6^ addressed the fuzzy hybrid flow-shop problem (FHFS) in an apparel manufacturing process. A parallel greedy algorithm (PGA) was proposed for solving the FHFS scheduling problem with lot sizes. Tliba et al.^7^ studied a dynamic scheduling problem of a real hybrid flow shop considering the specific constraints of a perfume manufacturing company. To improve the solving accuracy of flow shop scheduling problems, Liang et al.^8^ developed a computationally efficient optimization approach combining NEH and niche genetic algorithm (NEH-NGA). Shen et al.^9^ studied a permutation flow shop scheduling problem and established a chance-constrained programming model with faults. A hybrid genetic algorithm combined with asynchronous evolution was proposed to solve this model. To solve the hybrid flow-shop scheduling problem, Behnaz et al.^10^ developed a multi-objective robust mixed-integer linear programming (RMILP) model to accommodate the problem with the real-world conditions in which due date and processing time are assumed uncertain. Ilknur et al.^11^ integrated a random key genetic algorithm (RKGA) and technique for order preference by similarity to an ideal solution (TOPSIS) to compute makespan and solve the flow-shop scheduling problem (FSSP). Yu and Han^12^ examined machine scheduling problems that have been inspired by the production environment of semiconductor manufacturing, considering periodic machine maintenance. Yang^13^ studied a two-stage hybrid flow shop scheduling problem with time windows to minimize the total weighted completion times. Wang et al.^14^ investigated a multi-stage flexible flow shop scheduling problem with blocking on batch processing machines to minimize the makespan and the total energy consumption of machines simultaneously. Ren et al.^15^ developed a mixed integer programming model to solve flow-shop scheduling and investigated the optimal criteria, makespan and maximum delivery-completion time separately. Ali et al.^16^ presented a bi-objective mathematical model of a hybrid flow shop scheduling problem (HFSSP) with robots and fuzzy maintenance time. The proposed model minimized the mean completion time of jobs and the total cost of the processes based on maintenance costs and incurred transportation costs via robots. Ke et al.^17^ proposed an opposite artificial bee colony algorithm with opposition-based learning to solve the hybrid flow shop scheduling problem to minimize the makespan. Behnamian et al.^18^ studied a hybrid flow-shop scheduling problem in which the machining time of the workpiece varies with the machining speed and resource assignment of the machine. Chen et al.^19^ studied a flexible flow-shop scheduling problem with non-equivalent parallel machines considering bottleneck stations and proposed a heuristic machine selection rule based on bottleneck stations and a scheduling rule based on bottleneck stations to minimize the maximum completion time. Jitti et al.^20^ studied the scheduling problem of a flexible flow shop with non-equivalent parallel machines, and proposed a scheduling model with the dual objectives of minimizing the maximum completion time and minimizing the number of delayed workpieces, and solved the model with integer programming methods, heuristic rules and intelligent algorithms, respectively. Ceyda et al.^21^ studied the multi-task hybrid flow-shop scheduling problem. To reduce the computational complexity of the problem, an improved genetic algorithm with a novel crossover mechanism was proposed, and the effectiveness of the novel crossover mechanism was verified by case study. At the same time, the effectiveness of the improved genetic algorithm was also better than the tabu search algorithm. Reza et al.^22^ studied the flexible flow-shop scheduling problem with task blocking and no intermediate buffers and used the modal algorithm to solve it, and introduced the local search mechanism of nested neighborhood search in order to improve the search quality. Naderi et al.^23^ studied a hybrid flow-shop scheduling problem with machining adjustment time related to the machining start-up sequence and considering the workpiece handling time, and solved it by an improved simulated annealing algorithm with the objectives of minimizing the total completion time and minimizing the total delay time. Chen et al.^24^ studied the flexible flow-shop scheduling problem with non-equivalent parallel machines and bottleneck stations. To minimize the total tardiness, a bottleneck station heuristic algorithm considering EAAM rule, ECAM rule and ECALLM rule was proposed to solve the problem.

Regarding the assembly production scheduling issues of large complex equipment such as aircraft horizontal tails, aircraft, steam turbines, and ships, as well as the application of dynamic models in assembly workshop scheduling, numerous scholars have conducted comprehensive research. Zhong et al.^25^ studied the scheduling problem of aircraft assembly pulse production lines, aiming to reduce labor load as the optimization objective. They modelled the aircraft assembly pulse production line scheduling problem as two Markov decision processes, generated an approximate scheduling scheme for aircraft assembly through dual reinforcement learning agent decision-making, and utilized domain knowledge transfer methods to obtain a scheduling scheme with excellent comprehensive performance. Yu et al.^26^ studied the aircraft assembly scheduling problem considering material constraints. They took into account the team constraints, equipment constraints, process constraints, and material arrival constraints in actual production. With minimizing the maximum completion time as the objective function, they made decisions on team allocation, equipment allocation, and processing sequence for aircraft assembly tasks. They established a corresponding mathematical programming model and solved it using heuristic rules and variable neighborhood search algorithms. Bao et al.^27^ considered the aircraft assembly scheduling and resource allocation problem with variable working hours. To solve this problem, they proposed a selection strategy based on a genetic algorithm framework, which effectively addressed the related issues. Zhong et al.^28^ addressed the issue of dynamic modelling in aero-engine assembly production and proposed a genetic algorithm for cycle-based static scheduling optimization, tailored to the operational characteristics of aero-engines. Cai et al.^29^ studied a distributed assembly hybrid flow shop scheduling (DAHFS) problem with fabrication, transportation and assembly, and proposed a novel shuffled frog-learning algorithm with Q-learning (QSFLA) to minimize makespan. Zhao^30^ studied the aircraft assembly scheduling problem considering dynamic disturbances. Based on the ant colony algorithm, he established an integrated optimization model for the scheduling of assembly resources, defined the mapping relationship between construction graph paths and assembly operation cycles, provided the basic steps for mapping assembly operation cycles, and achieved resource scheduling optimization by optimizing artificial ant paths. Gao et al.^31^ proposed a dynamic scheduling framework for complex assembly workshops based on digital twins. By monitoring production interference events in real-time (such as order insertion and worker absences), a dynamic integer programming model was established, and an improved multi-objective evolutionary algorithm (IMOEA) was designed to optimize the maximum completion time and scheduling stability. Johnson et al.^32^ proposed a Multi-Agent Reinforcement Learning (MARL) framework for the dynamic scheduling problem of robotic assembly units, utilizing a distributed decision-making mechanism and incorporating the Double DQN algorithm to optimize real-time task allocation. Hu et al.^33^ investigated a flexible assembly workshop scheduling problem with dynamic product arrival, aiming to minimize total tardiness. They constructed a mathematical programming model based on event points, encompassing four decision sequences: processing machine allocation, processing operation sequencing, assembly station allocation, and assembly operation sequencing. Additionally, they proposed a deep reinforcement learning algorithm based on multi-agent systems for solving this problem. Wang et al.^34^ proposed a dynamic scheduling framework for assembly resources based on digital twins. By sensing equipment status and production disturbances (such as order insertion and resource failures) in real-time, a dynamic integer programming model was constructed, and an improved multi-objective evolutionary algorithm was combined to optimize scheduling stability and completion time. This framework has demonstrated dynamic response capabilities in an aircraft manufacturing case, resulting in a 15% increase in resource utilization. Hu et al.^35^ developed a Collaborative Co-Evolutionary Mathematical Algorithm (CCMA) for the scheduling problem of reconfigurable machine flexible assembly workshops. By decomposing the Mixed Integer Linear Programming (MILP) model and employing a dual-cooperation strategy, the algorithm effectively balances machine allocation and additive manufacturing module selection, achieving a performance improvement of 12.63% over traditional algorithms in 720 extended instances. Zhang et al.^36^ studied the flexible assembly workshop scheduling problem in a dynamic manufacturing environment. They proposed a joint model of constraint programming and mixed-integer linear programming in a dynamic environment, incorporating a part sharing mechanism and real-time scheduling rules (such as the “earliest completion time rule”), which significantly reduces the risk of delays in complex assembly tasks. Meng et al.^37^ addressed the issue of demand uncertainty in mixed-model assembly lines by proposing a robust mixed-integer linear programming model and developing a novel differential evolution algorithm based on Q-learning (QL-DE). This algorithm utilizes population-based evolutionary operators, within-population crossover operators, six task-centered and three product-centered neighborhood exploration operators, as well as reinforcement learning-based strategies, enabling the QL-DE algorithm to adaptively handle uncertain demands while optimizing the assembly line process. Gaber et al.^38^ investigated the integration of quantity, quality, and maintenance challenges in a serial multistage manufacturing system, jointly modelled buffer allocation and preventive maintenance, and reduced the probability of production interruptions through dynamic rescheduling strategies. The proposed model formulated a stochastic mixed integer nonlinear programming problem, and a comprehensive solution approach was employed, combining a genetic algorithm as the primary generative method with hybrid genetic algorithm variations. Renna^39^ investigated the buffer allocation issue in dynamic production scheduling, proposing a dynamic control method for buffer levels and the interval between two consecutive preventive actions. Additionally, an adaptive strategy for buffer capacity and maintenance actions was introduced, reducing the bottleneck process blockage rate by 30% in unreliable production lines. This provided theoretical support for the flexible allocation of resources in dynamic scheduling.

**Table 1:** The main contribution of the past works and this paper.

Authors and year	Main contributions	Algorithms/methods	Application scenario/specific issue
Rakrouki et al. (2023)	Investigated the two-machine permutation flow shop scheduling problem considering machine downtime	Deterministic Scheduling Model	General flow-shop
Devi et al. (2022)	Proposed a hybrid adaptive firefly algorithm to optimize multi-objective FJSP problem	Hybrid adaptive firefly algorithm	FJSP problem
Mousavi et al. (2018)	Investigated the multi-stage hybrid flow shop scheduling problem with dual additional characteristics	Hybrid heuristic algorithm	HFSP problem
Ceyda et al. (2005)	Proposed an improved genetic algorithm with a novel crossover mechanism	Improved genetic algorithm	Multi-task HFSP problem
Tliba et al. (2023)	Proposed a digital twin-driven dynamic scheduling method for hybrid flow-shop	Dynamic integer programming and multi-objective evolutionary algorithms	HFSP problem
Zhong et al. (2024)	Proposed a knowledge transfer method based on deep reinforcement learning	Deep reinforcement learning	Aircraft assembly pulse production line
Hu et al. (2024)	Proposed A collaborative evolutionary mathematical algorithm to address the scheduling issues in flexible assembly workshops for reconfigurable machines	Mixed integer linear programming (MILP)	Reconfigurable assembly workshop
Meng et al. (2025)	Explored the complexities of balancing and sequencing in mixed-model assembly lines under conditions of uncertain demand	Q-learning-inspired differential evolution algorithm	Mixed-model assembly lines
This paper	Established a static scheduling model for aircraft horizontal tail assembly, considering dynamic disturbances and artificial time uncertainties, and proposed an improved bi-level genetic algorithm (IBGA), integrating variable neighborhood search with elitist retention strategies	IBGA with VNS	FFSP problem with dynamic processing times

As shown in Table 1, at present, for the static scheduling of the flexible flow-shop problem, most scholars have solved the problem by establishing the FFSP static scheduling model and using intelligent algorithms such as genetic algorithm and simulated annealing algorithm, but there was less research on the scheduling problem of flexible flow shop for complex products such as aircraft flat-tail, especially for scheduling with dynamic processing times because of temporal fluctuations arising from human factors. Therefore, it is of great necessity to study the static scheduling problem of aircraft flat-tail assembly production.

To solve the aircraft flat-tail assembly production scheduling problem effectively, this paper establishes a static scheduling model for aircraft flat-tail assembly production, taking into account the constraints of the assembly process and frame occupation in the process of aircraft flat-tail assembly production, and minimizing the maximum delay penalty cost as the scheduling objective. Meanwhile, considering the group evolutionary search capability of the genetic algorithm and the local optimization improvement capability of variable neighborhood search, this paper proposes an improved bi-level genetic algorithm based on a two-stage genetic algorithm and variable neighborhood search, which can better achieve the balance of global search and local search, to achieve an effective solution of the aircraft flat-tail assembly production scheduling problem.

## Static scheduling model for flat-tail assembly production

### Problem description

The aircraft flat-tail assembly production static scheduling problem can be described as follows: the aircraft flat-tail assembly workshop is a flow workshop, each assembly task passes through each assembly station in turn, each assembly station has at least one equivalent parallel frame, each frame can be dynamically adjusted according to the different assembly tasks; the aircraft flat-tail assembly workshop is responsible for the assembly of a certain type of aircraft flat-tail products of various configurations, the assembly process is the same for different configurations of aircraft flat-tails, and they all need to go through each assembly process in turn, but the assembly time at the same assembly station is different; the assembly time of each assembly process, influenced by worker efficiency, follows a normal distribution around the mean assembly time, while the average worker efficiency on the same assembly frame fluctuates within a defined range; there is an assembly process constraint between multiple assembly processes of the same aircraft flat-tail, and the subsequent assembly process can only start after the completion of the previous process.

The static scheduling of aircraft flat-tail assembly production aims to minimize the total delay penalty cost of orders, and reasonably determine the arrangement of each assembly frame and the production sequence of each configuration assembly task.

**Fig. 1:**
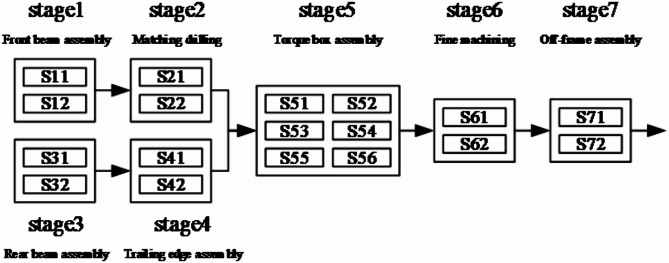
Flat-tail assembly flow diagram of a type of aircraft.

Take the flat-tail assembly and production of a certain type of aircraft in an aircraft manufacturer in Shanghai as an example, and its assembly flow diagram is shown in Fig. 1.

In order to facilitate the establishment of the static scheduling model for aircraft flat-tail assembly, the following assumptions are made in the scheduling model of this paper, while ensuring the actual characteristics of flat-tail assembly to the greatest extent possible: (1) at the initial moment, all orders arrive at the same time, and all tasks are released at the same time, and all assembly frames are available; (2) each assembly frame can only be available for one flat-tail at the same time; (3) the aircraft flat-tail assembly buffer capacity between adjacent assembly stations is unlimited; (4) the same flat-tail can only be assembled on at most one assembly frame at the same time; (5) during the assembly of the flat-tail, no assembly frame seizure is allowed; (6) once the assembly of the flat-tail is started, no interruption is allowed until the assembly is completed before leaving the assembly frame; (7) the handling time of the flat-tail between different assembly frames and the adjustment time on the frame is included in the assembly time.

### Mathematical model

For the convenience of description, the parameter variables, objective functions and constraints required for the FFSP problem are first defined, and the detailed variable names and their meanings are as follows.

Index.

*i* is the index of assembly tasks, *i* = *1*,*2*,*…*,*I*, *I* is the total number of assembly tasks.

*j* is the index of assembly process, *j* = *1*,*2*,*…*,*J*, *J* is the total number of assembly processes.

*x* is the index of the assembly frames, *x* = *1*,*2*,*…*,*X*_*j*_, *X*_*j*_ is the total number of assembly frames per assembly station.

Parameters and variables.

*T*_*ij*_ is the assembly time of assembly task *i* at assembly process *j*.

*u*_*ij*_ is the mean assembly time of assembly task *i* at assembly process *j*.

$\:\sigma\:$_*ij*_ is the standard deviation assembly time fluctuations of assembly task *i* at assembly process *j*, reflecting the time deviation caused by human operational interference.

*D*_*i*_ is the delivery time for assembly task *i*.

*C*_*i*_ is the assembly completion time for assembly task *i*.

*ST*_*ijx*_ is the start time of assembly process *j* of assembly task *i* on assembly frame *x*.

*FT*_*ijx*_ is the finish assembly time of assembly process *j* of assembly task *i* on assembly frame *x*.

*R*_*ijx*_ is the recovery time of assembly process *j* of assembly task *i* on assembly frame *x* due to dynamic interference.

*T*_*ijx*_ is the assembly time of assembly process *j* of assembly task *i* on assembly frame *x*.

*E*_*ijx*_ is the workers’ average efficiency index of assembly process *j* of assembly task *i* on assembly frame *x*.

*P*_*i*_ is the unit time delay penalty costs for assembly task *i*.

*w*_*j1j2*_ is the a 0/1 variable indicating the assembly process hierarchy constraint, with 1 indicating that assembly process *j*_*1*_ is assembled before assembly process *j*_*2*_.

*X*_*ijx*_ is the a 0/1 variable, 1 indicating that assembly process *j* of the assembly task *i* is assigned to assembly frame *x*.

*X*_*ghx*_ is the a 0/1 variable, where 1 means that assembly task *g* is assembled on assembly frame *x* before assembly task *h*.

Objective function and constraints.

1 $${f_1} = \min \sum\limits_{i = 1}^I {{p_i} \cdot \max \{ {C_i} - {D_i},0\} }$$

2 $$\mathop {FT}\nolimits_{{ijx}} =\mathop {ST}\nolimits_{{ijx}} +\mathop T\nolimits_{{ijx}} +\mathop R\nolimits_{{ijx}} ,\forall i,j,x$$

3 $${X_{ghx}} + {X_{hgx}} \le 1,\forall g,h \in i,\forall x$$

4 $${X_{ij{x_2}}} + {X_{ij{x_1}}} \le 1,\forall {x_1},{x_2} \in x$$

5 $$F{T_{ij{x_1}}} \cdot {w_{j1j2}} \le S{T_{ij{x_2}}} \cdot {w_{j1j2}},\forall i,x \cdot \forall {j_1},{j_2} \in j$$

6 $$\mathop T\nolimits_{{ij}} \sim N(\mathop \mu \nolimits_{{ij}} ,\mathop \sigma \nolimits_{{ij}}^{2} ),\forall i,j$$

7 $$\mathop E\nolimits_{{ijx}} \in (0,1],\forall i,j,x$$

8 $$\mathop T\nolimits_{{ijx}} =\mathop T\nolimits_{{ij}} /\mathop E\nolimits_{{ijx}}$$

Equation (1) is the objective function of the FFSP static scheduling problem, which indicates the minimization of the maximum total delay penalty cost. Equation (2) to (8) are constraints. Equation (2) represents the assembly time constraint, which means that the assembly completion time of each assembly process of each assembly task is equal to the sum of the assembly start time, the assembly time and the recovery time. Equation (3) represents the assembly frame occupancy constraint, which means that each assembly frame can only be available to one aircraft flat-tail at the same time. Equation (4) represents the assembly task occupancy constraint, which means that the same aircraft flat-tail can only be assembled on at most one assembly frame at the same time. Equation (5) represents the assembly process constraint, which means that the subsequent assembly process can only be started after the completion of the previous process. Equation (6) represents the assembly time follows a normal distribution around the mean assembly time; Eq. (7) represents the workers’ average efficiency index should be between 0 and 1; Eq. (8) represents the actual assembly time equals the value of the assembly time divided by the workers’ average efficiency index.

## Improved Bi-level genetic algorithm

The aircraft flat-tail assembly production static scheduling problem is an integrated problem of flow-shop scheduling and parallel machine scheduling, which consists of two main steps in the solving process: (1) the assignment of assembly frames at each assembly stage; (2) the ordering of assembly tasks on each assembly frame. The FFSP problem is a typical NP-hard problem, and Gupta^40^ has shown that even the FFSP with only two processes is an NP-hard problem. For the aircraft flat-tail assembly production scheduling, the assembly process is characterized by many assembly processes, many parallel assembly frames, many assembly tasks for multi-configuration products, uncertain assembly time, uncertain manual interference and complex assembly process constraints, so the computational complexity of its solution is higher, and an efficient algorithm needs to be designed to solve it.

Genetic Algorithm (GA) is a parallel search algorithm with the advantages of strong global search capability, high solution efficiency and good robustness. In order to better solve the two sub-problems of assembly frame assignment and assembly task sequencing, this paper adopts the bi-level Genetic Algorithm (BGA) to encode the assembly process and assembly frame respectively, and designs a bi-level crossover method for the encoding of the assembly process and frame.

Variable Neighborhood Search (VNS) is an effective local search method. Variable Neighborhood Search is a process of systematically changing the set of neighborhood structures to expand the search range and obtain a local optimal solution, and then systematically changing the set of neighborhood structures again to expand the search range based on this local optimal solution to find another local optimal solution. Variable-neighborhood search is a process of choosing the best of the best, which can well go beyond the local optimum to obtain a higher-quality global optimum solution.

To combine the group evolution global search capability of the genetic algorithm and the local optimization improvement capability of variable neighborhood search, this paper proposes a hybrid algorithm combining the bi-level genetic algorithm and variable neighborhood search, namely Improved Bi-level Genetic Algorithm (IBGA), to better achieve a better balance of global and local search, thus to achieve an effective solution of the aircraft flat-tail assembly production scheduling problem.

The algorithm process can be seen as follows.

### Step 1

Initialize the parameters.

Initialize the parameters of the IBGA algorithm, including population size *popsize*, crossover probability *Cr*, mutation probability *Mr*, elite retention ratio *Rr*, and the maximum number of iterations *maxGen*.

### Step 2

Determine the encoding rules and decoding rules.

Encoding is the first problem to be solved when solving scheduling problems with a genetic algorithm, that is, the chromosome representation of scheduling solutions. The production scheduling of aircraft flat-tail assembly consists of two steps: one is to assign the assembly tasks to each assembly frame, and the other is to sort the assembly tasks assigned to each assembly frame. Therefore, a two-segment encoding method is proposed, where one segment is used to express the assembly process of all assembly tasks, and the other segment represents the assembly frame number corresponding to each assembly process, and a feasible solution to this scheduling problem can be obtained through this one-to-one mapping relationship between assembly process and assembly frame.

1. Encoding based on assembly process *A*_*p*_.

The segment is coded with real numbers and the chromosome length is the total number of processes, if there are *n* assembly tasks, each assembly task has *k* assembly processes, and the chromosome length of *A*_*p*_ is *n×k*. The serial number *i* indicates the *i-th* assembly task, and the *k*_*i*_*-th* occurrence of *i* indicates the *k*_*i*_ assembly process of the assembly task. For example, If a chromosome is [1,3,2,1,1,2,3,3,2], 1, 2, and 3 represent assembly tasks 1, 2, and 3 respectively, and the three 1’s in the chromosome represent in turn the 1st–3rd assembly process of assembly task 1.

(2) Encoding based on assembly frame *A*_*x*_.

The segment coding also adopts the real number coding, the chromosome length is also *n×k*, and the set {1,2,…,*k*} represents each assembly process of the aircraft flat-tail, let the set of candidate assembly frames corresponding to each assembly process be {*x*_*1*_, *x*_*2*_, …, *x*_*k*_}, the set of candidate assembly frames corresponding to the *i-th* assembly process is *x*_*i*_, *x*_*i*_ is expressed as {*x*_*i1*_, *x*_*i2*_, …, *x*_*ni*_}, then the assembly frame-based code *A*_*x*_ can be expressed as {*l*_*1*_,*l*_*2*_,…,*l*_*i*_,…,*l*_*n×k*_}, where *l*_*i*_ is an integer within [1,*ni*], which is the *l*_*i*_*-th* element *x*_*li*_ of the candidate assembly frame set *x*_*i*_ for assembly process *i*, that is the assembly frame corresponding to the corresponding assembly process.

Similar to the encoding process, the decoding process of the bi-level genetic algorithm consists of two steps: (1) assigning each assembly task to the assembly frame; and (2) determining the specific start time and finish time of each assembly task on the assembly frame. In this paper, an active greedy decoding approach is adopted, the decoding method that minimizes makespan is preferred while taking into account the assembly process constraints of each assembly task.

### Step 3

Generate the initial population.

The initial population of chromosomes with the number of *popsize* is randomly generated according to the FFSP static scheduling optimization model.

### Step 4

Adaptation value evaluation.

The adaptation value evaluation means that the quality of the chromosome is evaluated, and the adaptation value function is defined as the inverse of the cost of minimizing the maximum delay penalty, then the larger the adaptation value is, the better the quality of the chromosome solution is.

### Step 5

Selection and elite retention strategy.

Selection is the process of choosing the best and elimination of the worst, that is eliminating the individuals with poor adaptation values and retaining the individuals with better adaptation values generation by generation, to achieve optimization of the objective function. Before using the genetic operator, to avoid the genetic operator destroying the goodness of individuals in the current population, the elite retention strategy is adopted, in which some individuals with good characteristics are not involved in the genetic operator operation and are directly inherited by the next generation population. In this paper, the elite retention strategy of roulette is used. The roulette wheel method selects individuals based on the probability of their adaptation values to the total adaptation values of all individuals, ensuring that individuals with larger adaptation values have a greater probability of being retained, while also having a certain probability of including individuals with smaller adaptation values, and ensuring the diversity of the offspring population.

### Step 6

Two-stage crossover.

Crossover operation is the process of exchanging certain genes of two or more individuals in a population to produce new offspring individuals, and it is the core part of the genetic algorithm. Considering the two-stage genetic algorithm with two coding methods, this chapter designs crossover methods for assembly process-based encoding and assembly frame-based encoding respectively.

1. POX crossover based on assembly process encoding.

**Fig. 2:**
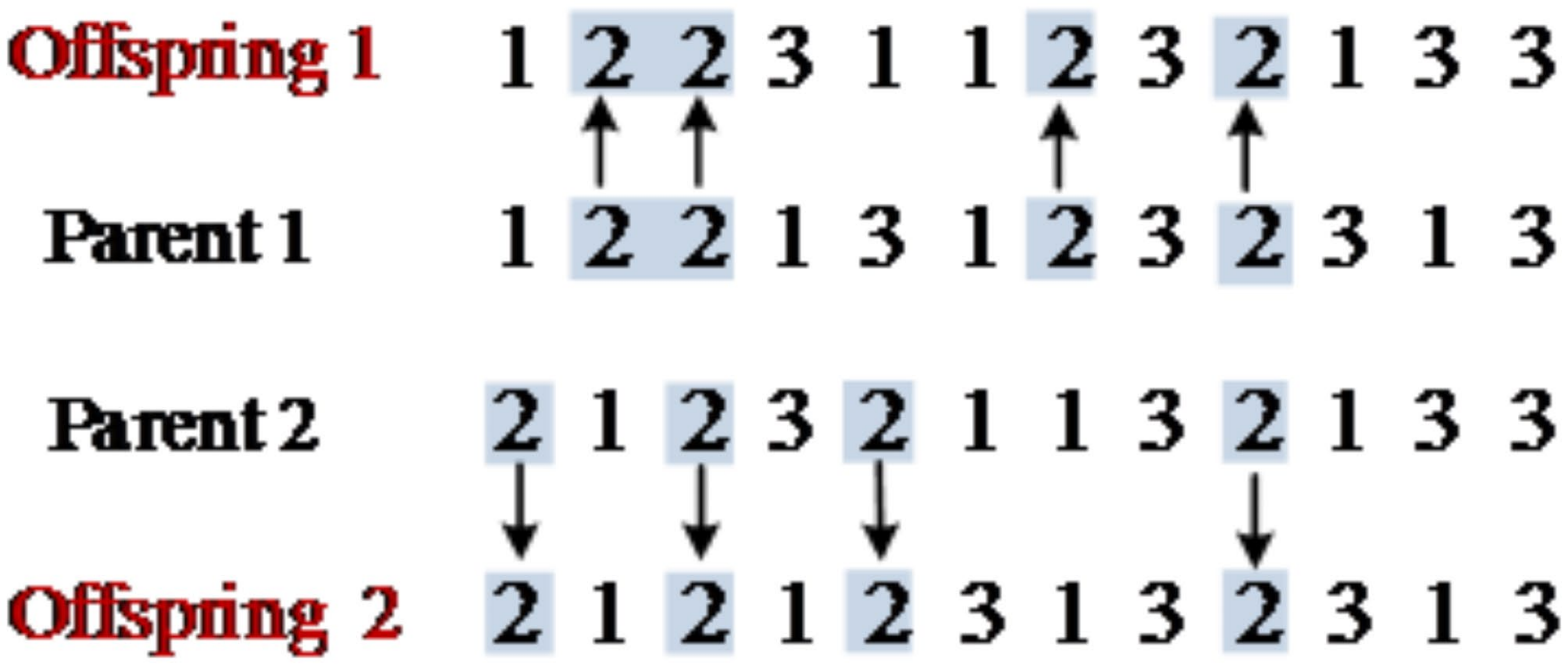
Schematic diagram of POX crossover based on assembly process encoding.

Firstly, all assembly processes are randomly divided into two sets *M*_*1*_ and *M*_*2*_; then, offspring 1 and offspring 2 inherit the *M*_*1*_ part from parent 1 and parent 2 respectively, and keep the position of *M*_*1*_ in the original parent chromosome; finally, the remaining gene parts of chromosomes of offspring 1 and offspring 2 are filled with the *M*_*2*_ chromosome parts from parent 2 and parent 1 respectively, and keep their order in the original parent chromosome. The specific crossover procedure is shown in Fig. 2.

(2) Multi-point crossover based on assembly frame encoding.

**Fig. 3:**
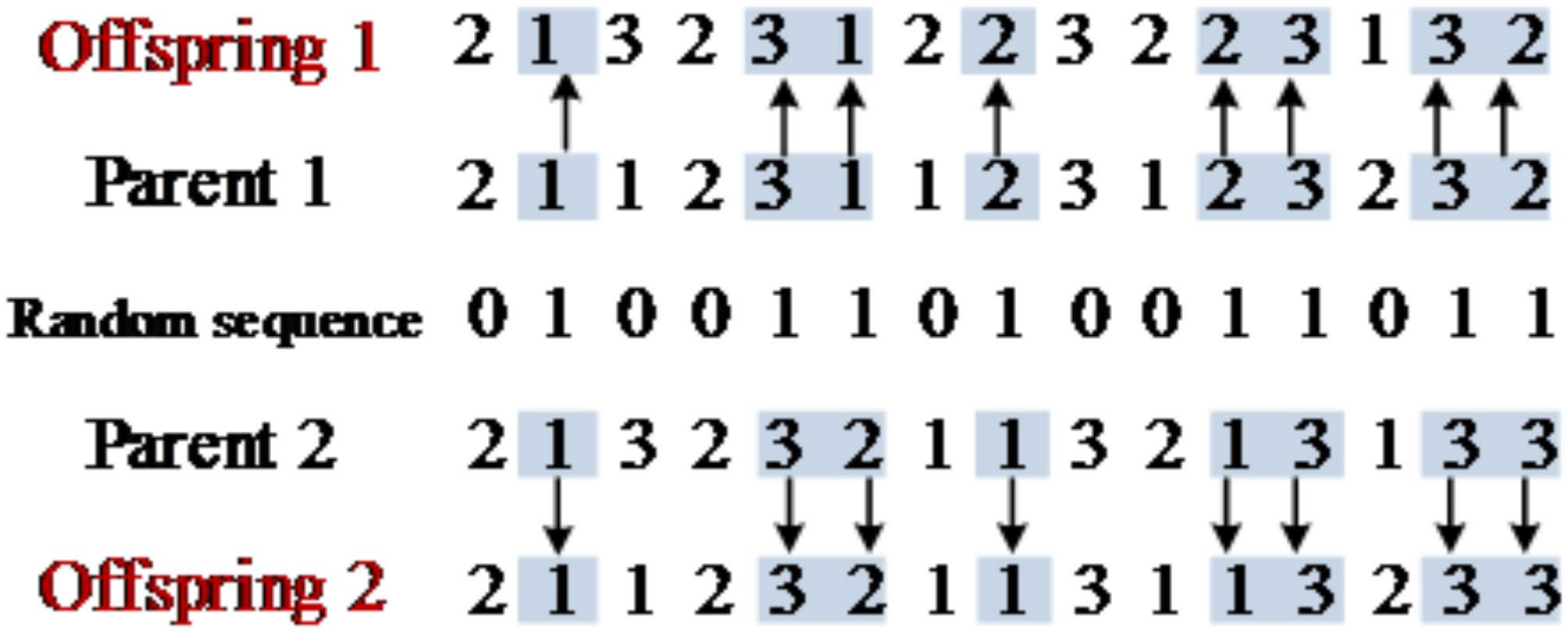
Schematic diagram of multi-point crossover based on assembly frame encoding.

The crossover based on assembly frame encoding firstly ensures that offspring 1 and offspring 2 are still viable solutions after each crossover, that means each gene of the chromosome encoded based on the assembly process still corresponds to the candidate assembly frame set for that assembly process, therefore, a multi-point crossover approach is proposed. Some genes of parent 1 and parent 2 are randomly selected and crossed in a one-to-one manner, which ensures that the offspring generated after each crossover are still viable solutions. The specific crossover operation procedure is shown in Fig. 3.

(3) Two-stage crossover based on assembly process encoding and assembly frame encoding.

When traditional genetic algorithms perform crossover operations, the children generated by the crossover are always accepted and inherited by the next generation, but this may destroy the better solutions already obtained due to the lack of a positive feedback mechanism in the genetic algorithm. Therefore, a two-stage offspring crossover model is proposed in combination with the two-segment encoding mechanism proposed in this chapter, different crossover counts are adopted for chromosomes encoded based on assembly processes and chromosomes encoded based on assembly frames, which can reduce a certain number of crossovers, reduce the complexity of the algorithm thus improving the solution efficiency, and ensure that the better offspring individuals have a higher probability of not being destroyed by crossover.

### Step 7

Mutation.

Mutation is used to ensure the diversity of the population and to prevent premature convergence of the genetic algorithm. For chromosome genes encoded based on assembly processes, random insertion variation is adopted, a gene is randomly selected and inserted into a random position; for chromosome genes encoded based on assembly frames, random assignment mutation is used, a gene is randomly selected and its value is replaced with another random assembly frame among its candidate assembly frame subset.

### Step 8

Variable neighborhood search.

Variable neighborhood search is an efficient local search strategy, and its neighborhood selection each time is a random process, which selects a random solution from the neighborhood and performs a local search on it. Aiming at the practical characteristics of the aircraft flat-tail assembly production scheduling problem and considering the solution quality and solution efficiency of the hybrid algorithm, three search neighborhoods are designed in this paper.

1. *N*_*1*_ *(S) Neighborhoods*: neighborhoods of assembly tasks on the same assembly frame.

- Random selection of an assembly frame *j*.
- Random selection of two assembly tasks *i*_*1*_ and *i*_*2*_ on assembly frame *j*.
- Exchange two assembly tasks *i*_*1*_ and *i*_*2*_.

**Fig. 4:**
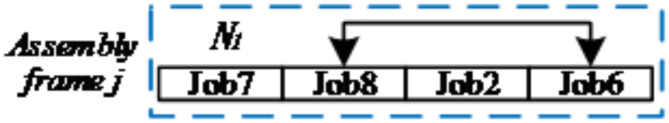
Diagram of neighbourhood structure-*N1(S)*.

The diagram of *N*_*1*_ *(S)* can be seen in Fig. 4.

(2) *N*_*2*_ *(S) Neighborhoods*: neighborhoods of assembly frames on different assembly frames.

- Random selection of two assembly frames *j*_*1*_ and *j*_*2*_ for the same assembly stage.
- Random selection of assembly task *i*_*1*_ on *j*_*1*_ and assembly task *i*_*2*_ on *j*_*2*_.
- Exchange two assembly tasks *i*_*1*_ and *i*_*2*_.

**Fig. 5:**
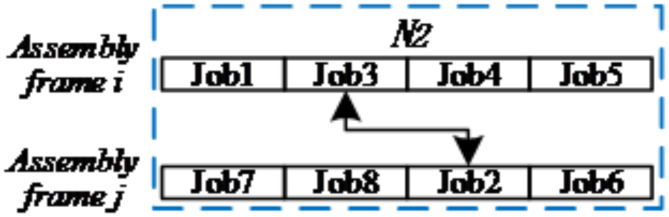
Diagram of neighbourhood structure-*N2(S)*.

The diagram of *N*_*2*_ *(S)* can be seen in Fig. 5.

(3) *N*_*3*_ *(S) Neighborhoods*: mixed assembly-frame and assembly-task neighborhood for one assembly frame and one assembly task.

- Randomly select an assembly task *i*_*1*_ and an assembly frame *j*_*2*_, and *i*_*1*_ does not belong to *j*_*2*_.
- Randomly select a reasonable location pos on *j*_*2*_.
- Transfer *i*_*1*_ to the pos position of *j*_*2*_ for assembly.

**Fig. 6:**
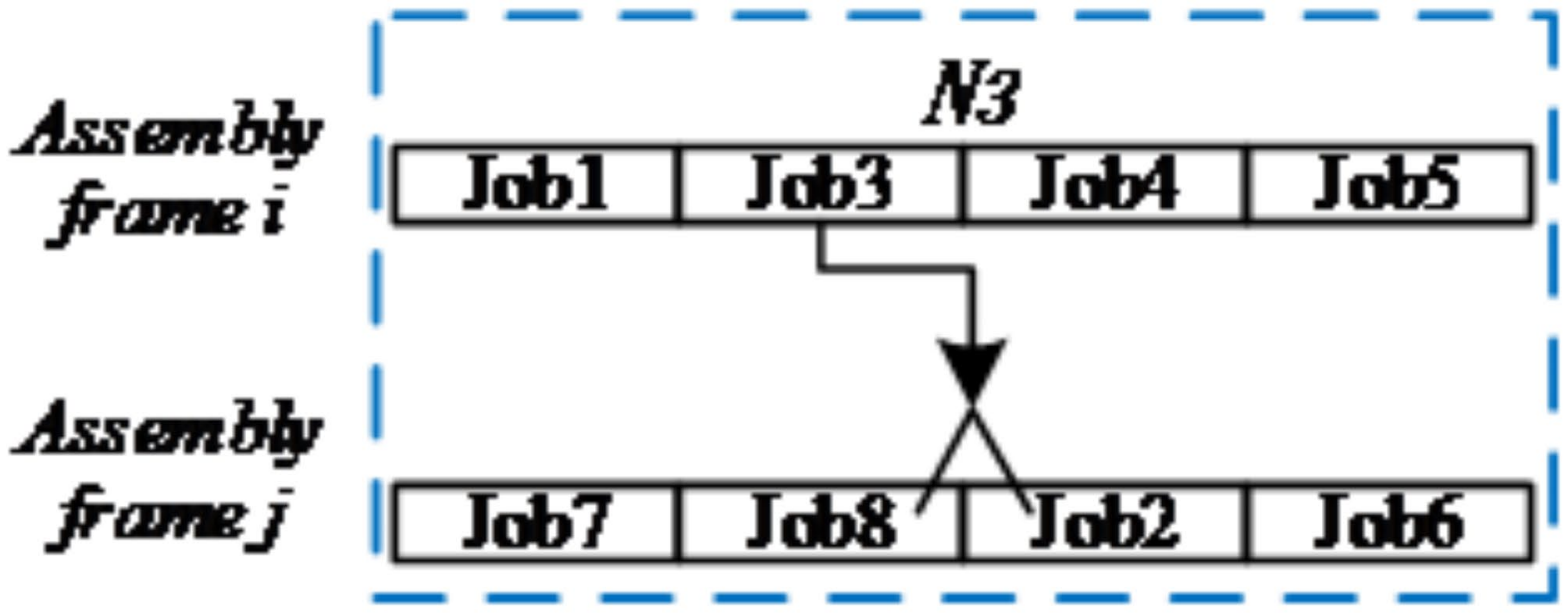
Diagram of neighbourhood structure-*N3(S)*.

The diagram of *N*_*3*_ *(S)* can be seen in Fig. 6.

**Fig. 7:**
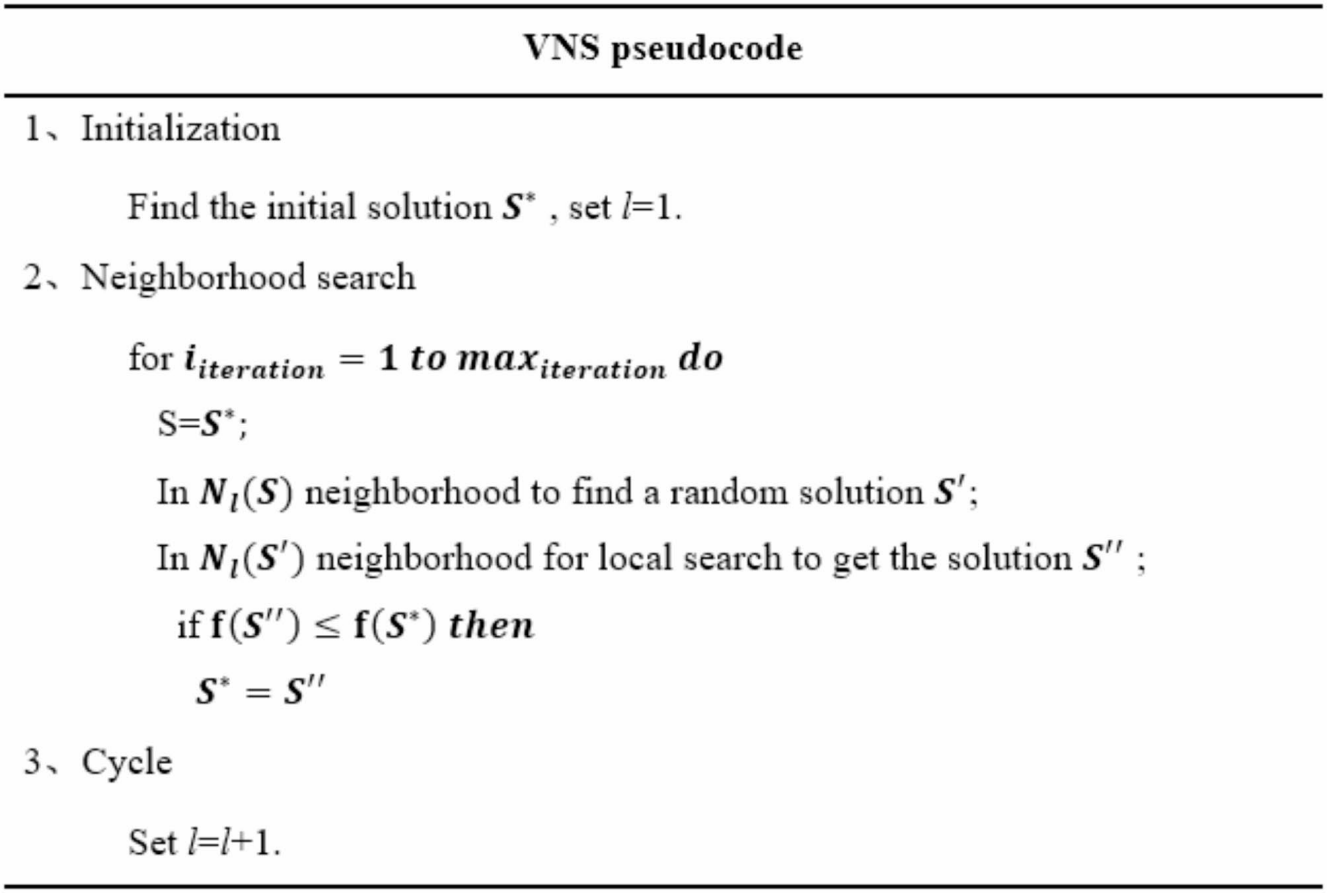
Variable neighborhood search pseudocode.

The pseudocode of variable neighborhood search is shown in Fig. 7.

### Step 9

*Gen* = *Gen* + 1, determine whether the termination condition is satisfied, if so, output the optimal solution; if not, return to step 4 and loop step 4 ~ step 8.

**Fig. 8:**
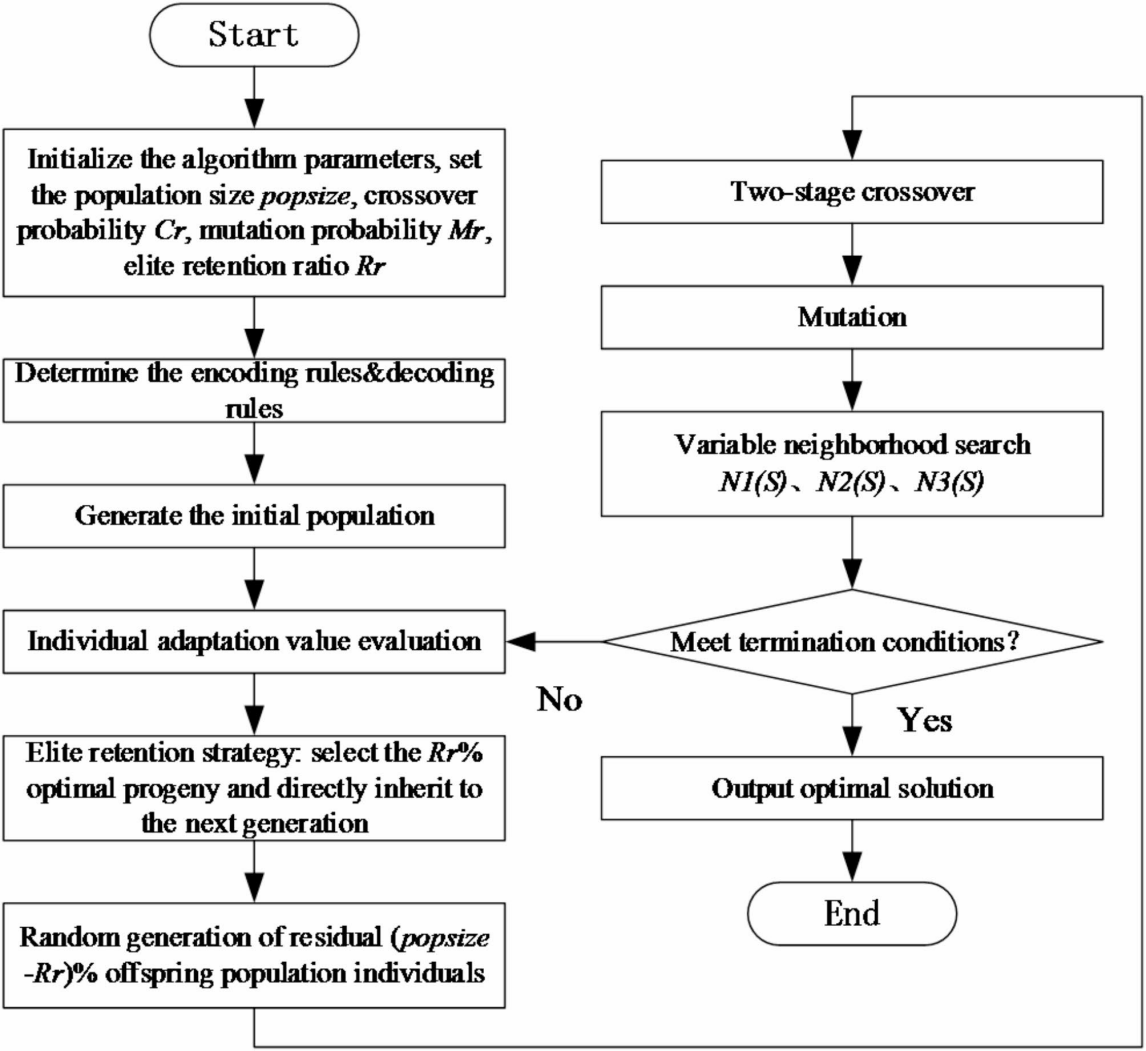
Flow chart of improved bi-level genetic algorithm.

The flow chart of the improved bi-level genetic algorithm is shown in Fig. 8.

## Experimental results and analysis

### Performance verification of improved bi-level genetic algorithm

For the FFSP problem, a validation case (shown in Table 2) was designed to verify the effectiveness of the improved bi-level genetic algorithm in solving the static scheduling problem of aircraft flat-tail assembly production by combining the characteristics of the number of assembly frames and the number of assembly processes in flat-tail assembly production, referring to the case generation method of Behnamian et al.^41^, and based on the actual data from the assembly plant of an aircraft manufacturing plant in Shanghai. The algorithm is programmed in C#, and the hardware environment is Core2 E8400 (2.26 GHz) with 4G memory and 32-bit Windows 7 operating system.

**Table 2:** Data of the validation case.

Parameters	Problem size		
	Small	Medium	Large
Number of assembly tasks	6	12	30
Number of assembly processes	4	8	12
Average assembly time	U[20,60]	U[20,60]	U[20,60]
Assembly worker efficiency	U[0.8,1]	U[0.8,1]	U[0.8,1]
The standard deviation of assembly time	U[1,3]	U[1,3]	U[1,3]
Average assembly recovery time	U[2,6]	U[2,6]	U[2,6]
Number of assembly frames	U[2,6]	U[2,6]	U[2,6]
Lead time for assembly tasks	U[100,1200]	U[300,1800]	U[600,2400]
Unit time delay penalty	U[2,5]	U[2,5]	U[2,5]

The solution results are significantly affected by the parameter settings of genetic algorithms. To efficiently select the parameter combinations for the IBGA algorithm, this paper employed orthogonal experimental design (OED) to test multiple parameter combinations. Through parameter adjustments, five important parameters and their corresponding ranges that significantly impact the experimental results were identified. Taking the medium problem size case for example, a 5-factor, 3-level experimental design table (as shown in Table 3) was designed to optimize parameters such as population size (*popsize*), crossover probability (*Cr*), mutation probability (*Mr*), number of encoding mutations based on assembly processes (*n*), and number of encoding mutations based on assembly frames (*k*). A L18(3^7) mixed orthogonal table (as shown in Table 4) was generated by Python’s PyDOE library.

**Table 3:** Range of key parameters for medium problem size.

Experiment No.	Population size popsize	Crossover probability Cr	Mutation probability Mr	Number of crossovers based on assembly processes encoding *n*	Number of crossovers based on assembly frames encoding k
1	50	0.6	0.01	3	20
2	100	0.7	0.05	6	40
3	200	0.8	0.1	9	60

**Table 4:** L18(3^7) mixed orthogonal table parameter combination for medium problem size.

Experiment No.	Population size popsize	Crossover probability Cr	Mutation probability Mr	Number of crossovers based on assembly processes encoding *n*	Number of crossovers based on assembly frames encoding k
1	50	0.6	0.01	3	20
2	50	0.7	0.05	6	40
3	50	0.8	0.1	9	60
4	100	0.6	0.01	6	40
5	100	0.7	0.05	9	60
6	100	0.8	0.1	3	20
7	200	0.6	0.05	3	60
8	200	0.7	0.1	6	20
9	200	0.8	0.01	9	40
10	50	0.6	0.1	9	40
11	50	0.7	0.01	3	60
12	50	0.8	0.05	6	20
13	100	0.6	0.05	9	20
14	100	0.7	0.1	6	40
15	100	0.8	0.01	3	60
16	200	0.6	0.1	6	60
17	200	0.7	0.01	9	20
18	200	0.8	0.05	3	40

To balance solution quality and efficiency, as well as to conserve computational resources, considering the scale of the aircraft flat-tail assembly production scheduling problem, this paper defined the maximum number of iterations for the algorithm as *maxGen* = 200.

**Fig. 9:**
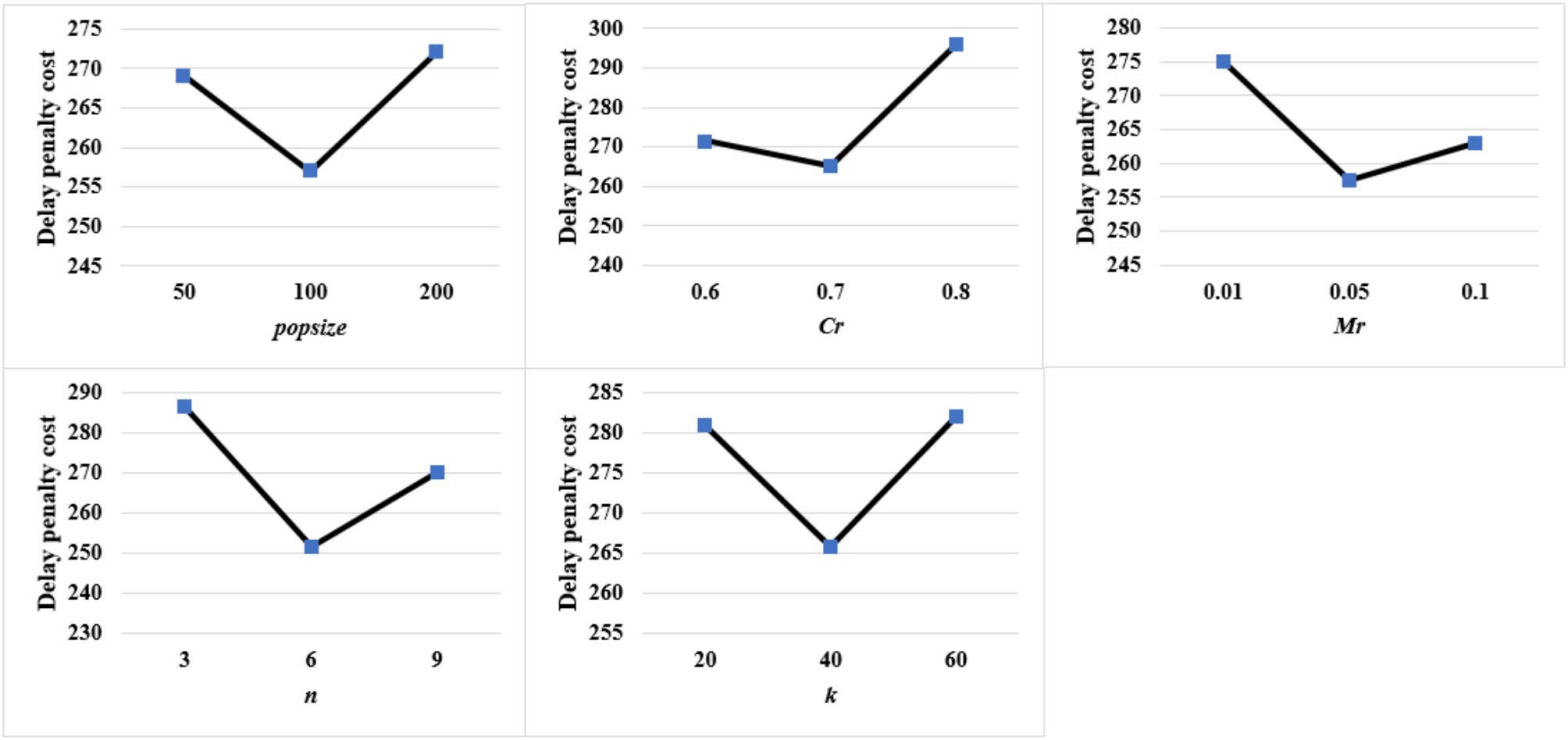
Mixed orthogonal experiment results table for medium problem size.

Based on the data presented in Table 2 and the mixed orthogonal parameter combination table mentioned above, with the objective of minimizing the maximum delay penalty cost, the parameters of the IBGA algorithm were optimized for medium problem scales. The orthogonal experimental results for partial key parameters are shown in Fig. 9. These results can further confirm the optimal combination of IBGA algorithm parameters. Repeating the same method, the optimal combination of key parameters for small and large problem scales can be calculated, as shown in Table 5.

**Table 5:** Optimal combination of key parameters for different problem sizes.

Problem size	Population size popsize	Crossover probability Cr	Mutation probability Mr	Number of crossovers based on assembly processes encoding *n*	Number of crossovers based on assembly frames encoding k
Small	50	0.7	0.05	3	20
Medium	100	0.7	0.1	6	40
Large	200	0.8	0.1	9	60

To verify the effectiveness and superiority of the improved bi-level genetic algorithm in solving the aircraft flat-tail assembly production scheduling problem, the data of the validation case in Table 2 are orthogonally combined according to the size of the problem, according to the number of assembly tasks of 6, 12, 30 and the number of assembly processes of 4, 8, 12, to obtain 9 sets of validation cases, the average assembly time of each case obeys the uniform distribution of U[20,60], the assembly worker efficiency obeys the uniform distribution of U[0.8,1], the Standard deviation of assembly time obeys the uniform distribution of U[1,3], the average assembly recovery time obeys the uniform distribution of U[2,6], the number of assembly frames obeys the uniform distribution of U[2,6], the unit time delay penalty cost (unit: thousand dollars per hour) obeys the uniform distribution of U[2,5], and the assembly task delivery time obeys the uniform distribution within each case according to the size of each case. The specific test cases are shown in Table 6. The experimental group 6 × 4 in the table indicates that there are 6 aircraft flat-tails to be assembled, and each aircraft flat-tail has 4 assembly processes.

**Table 6:** Data of the validation case.

Case number	Experimental group	Number of tasks	Number of processes	Delivery date	Other uniform data
1	6 × 4	6	4	U[100,200]	Average assembly time: U[20,60] Assembly worker efficiency: U[0.8,1] The standard deviation of assembly time: U[1,3] Average assembly recovery time: U[2,6] Number of assembly frames: U[2,6] Unit time delay penalty: U[2,5]
2	6 × 8	6	8	U[400,800]	
3	6 × 12	6	12	U[800,1200]	
4	12 × 4	12	4	U[300,600]	
5	12 × 8	12	8	U[600,1000]	
6	12 × 12	12	12	U[1200,1800]	
7	30 × 4	30	4	U[600,900]	
8	30 × 8	30	8	U[900,1200]	
9	30 × 12	30	12	U[1600,2400]	

In the actual production planning and scheduling of aircraft flat-tails, some simple rules are mostly used. At present, the more common is First In First Out (FIFO), which is a scheduling method that first sorts all assembly tasks according to the priority of delivery date, and at the first assembly station, the assembly is put into production on the frame in the order of the highest to lowest priority of delivery date. From the second to the last assembly station, the FIFO rule and the free assembly frame priority rule are adopted, and a scheduling solution is obtained when all assembly tasks are sorted. For the 9 test cases in Table 6, the improved bi-level genetic algorithm (IBGA), the FIFO rule, and the simulated annealing algorithm (SA) proposed by Naderi^42^ are solved to obtain the average value (*avg*) and the optimal value (*min*) of the delay penalty cost and the corresponding computation time for each algorithm.

The solution results are shown in Table 7. Analyzing the solution results, it can be seen that (1) in terms of solution quality, IBGA is significantly better than the other two methods in terms of optimal value and average value, SA has the second-best solution effect, and FIFO has the worst solution quality. (2) In terms of solution time, FIFO has the shortest solution time and the most efficient solution, followed by SA, while IBGA has a longer solution time. In terms of the absolute size of the solution time, for the assembly production scheduling problem of 30 aircraft flat-tails, the solution time is about 1 min, while for the aircraft flat-tail assembly production planning scheduling, the production plan is made monthly, thus the solution time of IBGA is fully acceptable. Therefore, the solution quality of IBGA is better than other algorithms, and the computation time can fully meet the requirements of aircraft flat-tail assembly production scheduling.

**Table 7:** Experimental results of test cases.

Experimental group	Delay penalty cost (/$1000)						Calculation time (/ms)		
	IBGA		SA		FIFO		IBGA	SA	FIFO
	Min	Avg	Min	Avg	Min	Avg			
6 × 4	28.6	34.7	28.0	37.3	58.9	80.2	163.8	137.0	111.8
6 × 8	157.1	185.6	173.3	214.3	355.6	459.2	1185.8	775.4	266.9
6 × 12	128.6	148.4	142.7	184.0	283.6	364.5	3067.9	2300.5	782.1
12 × 4	177.1	211.3	198.7	247.2	412.4	520.8	1156.1	638.5	344.2
12 × 8	347.1	387.7	360.0	431.2	658.9	846.4	4603.0	2931.3	1036.4
12 × 12	397.1	441.8	416.0	486.0	785.5	992.2	10170.4	7171.2	2565.8
30 × 4	250.0	271.7	244.0	288.8	501.8	652.7	14267.0	9633.3	6033.4
30 × 8	412.9	453.9	402.7	464.6	746.2	969.1	35651.1	24971.5	10099.2
30 × 12	448.6	481.7	445.3	509.3	802.9	991.7	214450.4	133999.4	35589.7

In order to intuitively compare the optimization results of solutions for different size cases together, this paper draws on the concept of Relative Percentage Deviations (RPD) proposed by Gholami^43^. The RPD calculation formula is shown in Eq. (9).

9 $$RPD=\frac{{\mathop {avg}\nolimits_{A} - min}}{{min}}$$

**Fig. 10:**
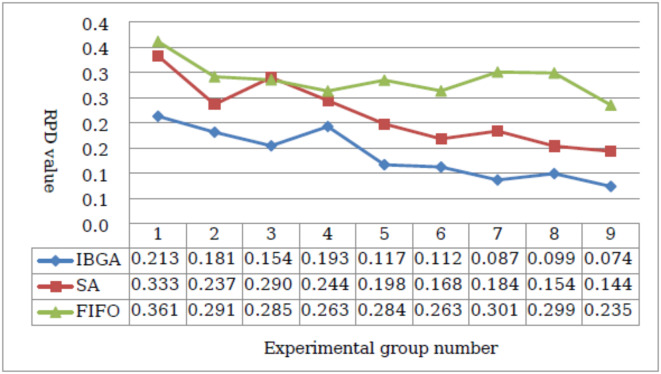
Comparison of the results of three static scheduling algorithms.

*Min* is the optimal value of the objective function obtained by the three algorithms in each case, and *avg*_*A*_ is the average value of each algorithm in the corresponding case. The smaller the RPD, the closer the average value of the algorithm is to the known optimal solution, and thus the better the algorithm’s effectiveness. As shown in Fig. 10, the horizontal coordinate is the number of the experimental group, the vertical coordinate is the RPD value of the three algorithms, and the detailed RPD values of the three methods are marked below the horizontal axis, the RPD value of IGBA is smaller than that of SA and FIFO, and thus its solving effectiveness is better than the other two algorithms.

### Case study

In this paper, the effectiveness of the improved bi-level genetic algorithm is verified by the actual production data of an aircraft manufacturing plant in Shanghai. An aircraft manufacturing plant in Shanghai undertakes the subcontracting business of flat-tail of a certain type of aircraft, which contains three types of configurations A, B and C, and a certain number of flat-tail assembly tasks for each configuration need to be completed every month. Currently, the assembly production schedule is mainly completed manually by the planner according to the FIFO rules, which results in the poor rationalization of the assembly schedule, the frequent delay of orders, and the low utilization rate of the assembly frame.

The rated assembly time for each configuration of flat-tail is shown in Table 8.

**Table 8:** Assembly time of each configuration of a particular flat-tail type.

Configuration	Assembly time (/0.5 day)						
	ACC200	ACC205	ACC210	ACC220	ACC235	ACCA240	ACC245A
A	6	4	6	8	5	3	6
B	4	3	5	6	4	3	4
C	3	4	4	5	3	3	4

To ensure the effectiveness of the example comparison results, the production planning data of the flat-tail assembly workshop from March to August 2024 was selected to compare with the solution results of the IBGA. The delivery plan for each configuration from March to August is shown in Table 9. The delivery method for each configuration is to deliver half of the required orders in the middle month and at the end of the month. The tardiness penalty cost is related to time, and the penalty cost for A, B, and C configurations are $4000, $3500, and $1600 (unit: aircraft/dollar/hour) respectively.

**Table 9:** Monthly assembly plan of each flat-tail configuration.

Configuration	Month of 2024 (/aircraft)					
	March	April	May	June	July	August
A	10	9	9	8	9	7
B	8	9	9	8	9	10
C	8	9	9	9	10	9
Total	26	27	27	25	28	26

The solution results of the IBGA and the actual production data are shown in Table 10.

**Table 10:** Comparison of the results of IBGA and the actual production data.

Month	Total tardiness cost (/dollar)		
	IBGA	Actual production data	Reduced proportion
March	11,560	13,600	15.00%
April	12,300	15,400	20.13%
May	14,200	16,200	12.35%
June	10,870	12,300	11.63%
July	15,490	18,900	18.04%
August	7900	8700	9.20%

**Fig. 11:**
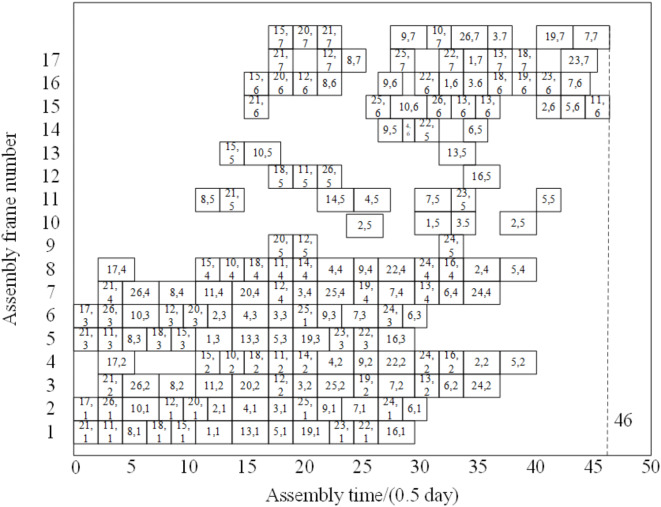
Gantt chart of aircraft flat-tail assembly production schedule in August 2024.

It can be seen from Table 10 that the adoption of the IBGA flat-tail assembly production static scheduling method reduces the flat-tail order delay penalty cost by 14.39% per month on average, with significant optimization effectiveness. Figure 11 shows the Gantt chart of the aircraft flat-tail assembly production schedule in August. The abscissa represents the assembly working hour with the unit of 0.5 days, and the ordinate represents the assembly frame number.

**Fig. 12:**
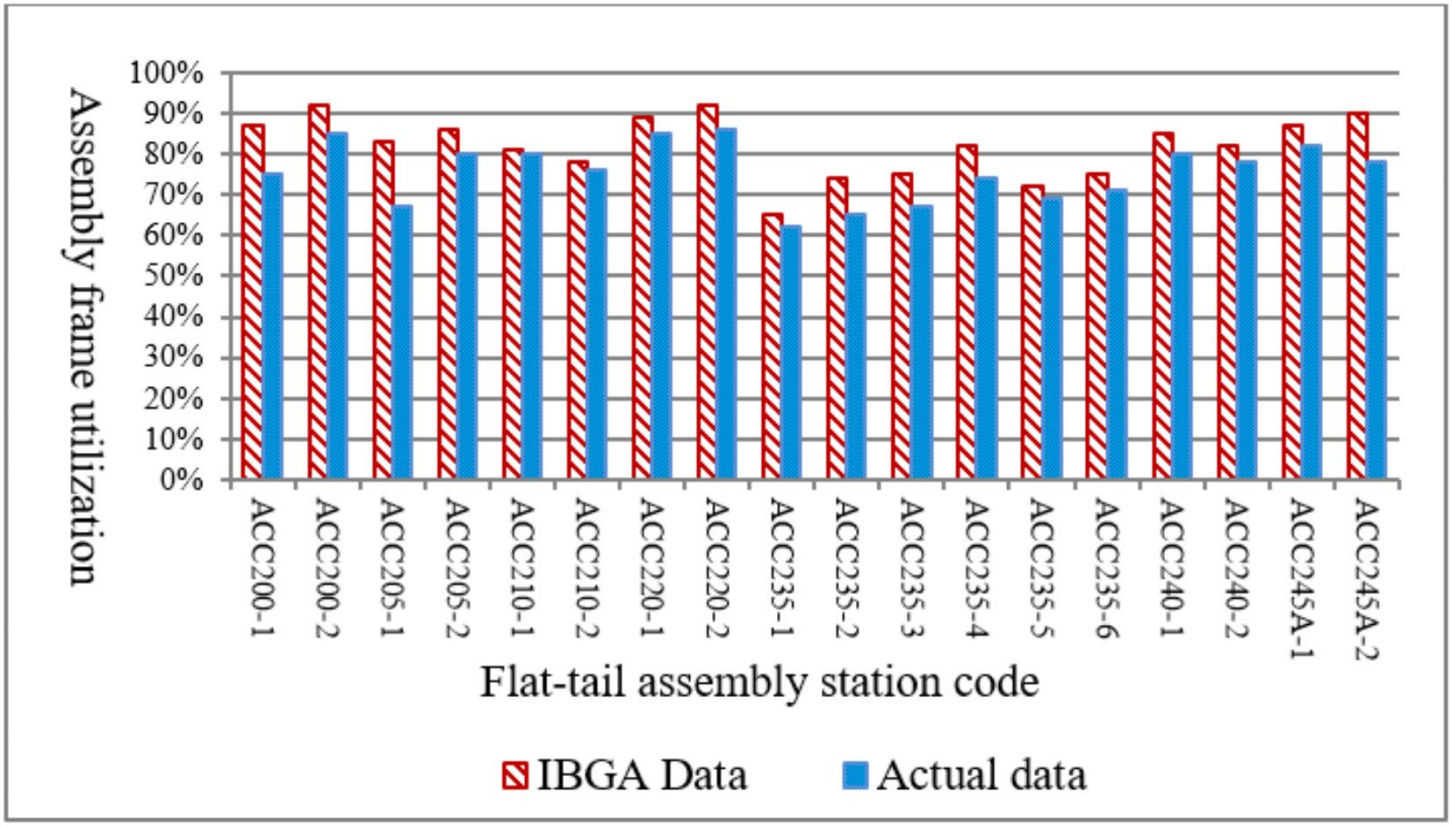
Comparison of assembly frame utilization before and after IBGA optimization.

Static scheduling of aircraft flat-tail assembly production based on the IBGA algorithm can significantly improve the utilization rate of assembly frames at each station. Taking the production data of aircraft flat-tail assembly in August 2024 as an example, as shown in Fig. 12, the average utilization rate of the assembly frame increased from 75.6 to 85.3% after adopting the IBGA algorithm, and the utilization rate of the assembly frame improved significantly.

The results of the case study show that the static scheduling method of aircraft flat-tail assembly production based on the improved bi-level genetic algorithm proposed in this paper has good application effects and can effectively guide the aircraft flat-tail assembly production, improve the utilization rate of the assembly frame and increase the delivery capability of the aircraft flat-tail.

## Conclusion

This paper presents a novel solution to the static scheduling problem in aircraft flat-tail assembly production. We developed a novel approach using an improved bi-level genetic algorithm (IBGA) that effectively addresses multiple assembly processes, parallel frames, and multi-configuration mixed-flow assembly challenges. The proposed method incorporates a two-stage genetic algorithm encoding scheme to handle assembly frame assignment and task sequencing, complemented by a variable neighborhood search method to prevent premature convergence.

The key contributions of this research are threefold: First, we formulated an FFSP static scheduling model aimed at minimizing maximum delay penalty costs. Second, we designed and implemented an improved bi-level genetic algorithm that efficiently solves the complex scheduling problem. Third, through extensive computational experiments and real-world case studies, we demonstrated that our approach successfully reduces flat-tail assembly cycle time, lowers delay penalty costs, and maintains high resource utilization rates.

The effectiveness of our proposed solution has significant implications for practical applications in aircraft assembly production scheduling. The results provide valuable insights for industry practitioners and establish a solid foundation for future research. Potential future directions include exploring dynamic scheduling capabilities for real-time disruption handling, integrating advanced technologies such as big data and deep learning for adaptive optimization, and extending the current model to address other complex assembly scenarios in the aerospace industry.

## Data Availability

The data that support the findings of this study are included in this article, and also available from the corresponding author upon reasonable request.
